# Obstructive Dysmenorrhœa and Sterility from Contraction of the Cervix Uteri

**Published:** 1859-09

**Authors:** 


*Obstructive Dysmenorrhœa and Sterility from Contraction of the Cervix Uteri.* By Prof. SIMPSON.

When dilatation is effected mechanically, whether slowly by sounds, or rapidly by sponge tents, relapse of the stricture or contraction is very apt to return after a time, just as so often happens after the treatment of bad stricture of the male urethra, by merely dilating instruments. The best and speediest means of cure is to have recourse at once to dilatation of the os by incising it at both sides. For the performance of this operation, an instrument or metrotome is required; this must be introduced as far as the os internum, where the incision begins, at first quite shallow, and then deeper as the instrument is withdrawn, till at the os externum the cervix is cut across in all its thickness. If you cut too deeply in the upper portion of the cervix, you run the risk of wounding some of the veins of the plexus uterinus—otherwise the hemorrhage is not of consequence. The wound must be opened up every two or three days with the finger to prevent union, or the corners of the wound may be touched with a piece of nitrate of siver, with a like good result. There are few operations in surgery "so perfectly simple in their performance so entirely satisfactory in their results."

